# Fabrication of an Oscillating Thermocycler to Analyze the Canine Distemper Virus by Utilizing Reverse Transcription Polymerase Chain Reaction

**DOI:** 10.3390/mi13040600

**Published:** 2022-04-12

**Authors:** Jyh-Jian Chen, Zong-Hong Lin

**Affiliations:** Department of Biomechatronics Engineering, National Pingtung University of Science and Technology, Pingtung 91201, Taiwan; o7392618@yahoo.com.tw

**Keywords:** RT-PCR, infectious diseases, oscillating thermocycler, canine distemper virus

## Abstract

The reverse transcription-polymerase chain reaction (RT-PCR) has been utilized as an effective tool to diagnose the infectious diseases of viruses. In the present work, the oscillating thermocycler is fabricated and performed to carry out the one-step RT-PCR process successfully. The ribonucleic acid (RNA) mixture is pipetted into the fixed sample volume inside an aluminum reaction block. The sample oscillates the pathway onto the linear motion control system and through the specific RT-PCR heating zones with individual homemade thermal control modules. The present oscillating thermocycler combines the merits of the chamber type and the CF type systems. Before PCR, the reaction chamber moves to the low-temperature zone to complete the RT stage and synthesize the complementary deoxyribonucleic acid (DNA). Next, the low-temperature zone is regulated to the annealing zone. Furthermore, the reactive sample is moved back and forth among three isothermal zones to complete PCR. No extra heating zone is required for the RT stage. The total length of the moving displacement of the chamber is within 100 mm. The miniaturization of the oscillating thermocycler can be expected. In our oscillatory device, the denaturation zone located between the annealing and extension zones is suggested as the appropriate arrangement of the heating blocks. Heat management without thermal cross-talk is easy. Finally, an improved oscillating device is demonstrated to execute the RT-PCR process directly, utilized to amplify the canine distemper virus templates successfully, which could be well applied to a low-cost DNA analysis system in the future.

## 1. Introduction

Using the culture method in the isolation of pathogenic viruses is a conventional way to determine the viral infection. Other tools, such as immunological and serological methods, are occasionally utilized for the detection of the virus antigen and/or antibody. These kits provide early detection and easy interpretation. However, most of these methods are some limitations concerning the specificity and sensitivity or sometimes give false-negative results. So, a rapid, accurate, precise, specific, and sensitive analysis is required for the diagnosis of viral infection in the clinics.

Over the past decades, the developments of molecular techniques have revealed their suitability for pathogenic diagnosis. After a technique of in vitro deoxyribonucleic acid (DNA) amplification with a thermostable DNA polymerase was introduced, it has been widely applied to the identification of several types of diseases, including viral infection. Polymerase chain reaction (PCR) is one of the most significant techniques that can exponentially amplify specific segments of DNA. The PCR process goes through the following stages. Firstly double-stranded DNA (dsDNA) is separated into two single-stranded DNA (ssDNA) at high temperatures (denaturation stage at about 95 °C or 368 K). Secondly, primers bind to their complementary site of the ssDNA at low temperatures (annealing stage at about 55 °C or 328 K). Thirdly, the thermostable DNA polymerase extends the primers, complementary to the DNA template at intermediate temperatures (extension stage at about 72 °C or 345 K). Three consecutive stages complete one PCR cycle, and each piece of target DNA in the mixture can be replicated. After finishing 30 or above cycles, a small number of DNA fragments are duplicated into a large number of sample products for detection.

Recently, reverse transcription PCR (RT-PCR) has been utilized as a successful tool to diagnose the infections of viruses [1,2,3]. Complementary DNA (cDNA) of the virus is synthesized from messenger ribonucleic acid (mRNA) by the RT reaction, and cDNA is utilized as the template for PCR and further viral detection. To perform the RT-PCR technique, a commercial thermocycler (also known as a PCR machine) is commonly used. A standard thermocycler consists of a controlled heating/cooling module and several reaction tubes [4]. Due to the high thermal mass of a thermocycler, a low thermal ramping rate is implemented, and a long reaction time is needed. During the last decades, the miniaturized reactor has become increasingly popular due to its small thermal mass and the large heat transfer rate inside the system [5]. Lee et al. [5] used the chip-based RT-PCR for the identification of influenza A and B viruses, parainfluenza viruses 1–4 (PIV1–4), human metapneumovirus, adenovirus, human rhinovirus, respiratory syncytial virus (RSV), and SARS-CoV-2 performed with the commercial equipment. Zhu et al. [6] presented an oil-covered silicon microchip system to achieve the RT-PCR assay. The feasibility of the system for gene expression analysis of mir-122 was demonstrated.

The conception of microfluidic technologies has made the miniaturization of analysis instruments a reality. There are some advantages, such as portability, shortened reaction time, and reduced reagent volume, to utilizing the PCR chips [7,8]. The design of miniaturized thermal cyclers can be categorized into the stationary chamber type and the continuous flow type. The stationary chamber-type devices are a scaled-down version of commercial thermocyclers. The reaction mixture is kept static, and the temperature of the reaction chamber is cycled. The stationary chamber-type devices perform repeated heating and cooling to accomplish the PCR process. Any enzyme adsorbed by the walls of the reaction volume is not obvious. The continuous flow PCR (CFPCR) devices can be realized by a time-space conversion of conventional PCR machines. By keeping the temperatures constant over time at different locations (ranging from one to three) in the device, the reaction mixture moves through the individual isothermal zones to finish the reaction stages. Not only is the residence time in which a sample is exposed to each isothermal region, but also the number of thermal cycles is determined by the arrangement of heating zones [9]. Each type has its own merits.

There are three main forms of the CFPCR reactors: unidirectional, closed-loop, and oscillatory reactors. In the unidirectional reactor, the reactive sample flows through different isothermal zones to accomplish several PCR cycles along one tube or channel. Felbel et al. [10] presented a flow-through RT-PCR micro-reactor. The device carried out the detection of the human papillomavirus (HPV) 16 DNA genome and viral oncogene transcripts, respectively. Li et al. [11] developed a microfluidic device that integrates stationary RT and CFPCR with the fluorescence detection of rotavirus. The experimental arrangement included the syringe pump for compelling the reaction sample flowing continuously through one polytetrafluoroethylene (PTFE) tube and the fluorescence microscope for product detection. Pham et al. [12] fabricated an RT-PCR microdevice operated with a portable pump for sample delivery, a single heater for temperature control, and a downstream fluorescence module for detection. The device was assembled by wrapping a PTFE tube around a polycarbonate mold for both the RT of RNA and the PCR of cDNA for the detection of the 154 bp *ACTB* gene. The cycling number is fixed during unidirectional CFPCR, and the DNA adsorption onto the inner surface is unavoidable because of the long length of the tube or channel. Usually, an extra heating zone for the RT stage is needed.

In the closed-loop reactor, the reactive sample passes the different isothermal zones and revolves around the closed pathway within one PCR cycle. You et al. [13] used the wire-guide droplet method for rotating the solution droplets through some isothermal zones. With the RT-PCR system, 160 bp gene sequences were amplified from 2009 H1N1 influenza A. Qiu et al. [14] developed convection PCR in a capillary tube for the diagnosis of influenza A (H1N1) virus. A temperature gradient across the tube, which is heated from the bottom end, generates a continuous circulatory flow for PCR. Some active valves for sealing the inlet and outlet have to be integrated into the device. In the oscillatory reactor, the reactive sample is moved back and forth among several isothermal zones. Prakash et al. [15] demonstrated a microdevice that is capable of conducting up to eight parallel RT-PCR reactions per usage. The oscillating actuation of PCR droplets was transported between two isothermal zones by utilizing a combination of electrostatic and electrowetting droplet actuation. Influenza A and B were accurately identified. Within the closed-loop and oscillatory reactors, the cycling number is flexible, and the number of biological molecules adsorbed onto the channel or tube can be greatly reduced due to the short moving distance of the sample.

Previous works regarding miniaturized RT-PCR devices have utilized contact heating apparatus, such as thin-film resistors [10,14,15], cartridge heaters [11], hot plates [12], printed circuit board (PCB) heaters [13], or non-contact heating device such as infrared sources [16,17]. For conventional contact heating in devices, the heat energy transferred from the heat sources is directed to highly thermally conductive metal blocks, then through the sample chamber, and finally into the DNA mixture [11]. By using non-contact infrared heating in devices, the energy heats the sample directly rather than has to heat the medium surrounding the chamber or the chamber itself. Saunders et al. [16] reported an RT-PCR system with an infrared laser system to accomplish PCR in the chamber. The system also consists of a microfluidic device featuring a 1 mL reaction chamber and a microscope for fluorescence detection. The comparison of important technical aspects with previous reports and the current work is demonstrated in the Supplementary Materials, Table S1.

Canine distemper virus (CDV) is a member of the genus *Morbillivirus* of the *Paramyxoviridae* family and causes an extremely contagious disease. CDV is spread by direct aerosol contact. The infection of CDV often induces a fatal illness in dogs, other carnivores, noncarnivores, as well as marine mammals [18,19]. Furthermore, a possible link between Paget’s disease of bone in humans and CDV infection was presented by epidemiological studies [20,21]. Though the live-attenuated vaccine against the disease caused by CDV has been used widely, the detection of the neutralizing antibodies against CDV is not fully reliable for diagnosis. It is because high neutralizing antibodies are existed inside many puppies with maternal antibodies and vaccinated dogs [22]. RT-PCR has been employed successfully for the diagnosis of CDV since 1999. The detection of even a few copies of viral RNA is particularly valuable for the identification of subclinically infected animals that contribute to the disease spread. So, a portable molecular analysis device for RT-PCR is essential for viral detection of CDV in clinics.

The design concepts of the oscillating thermocycler we use in the present work are the continuation of our previously presented bidirectional thermocycler [9,23]. The reaction chamber with the DNA mixture was driven by the moving stage, which is connected to the linear motor. The user subroutines coded and compiled by the FORTRAN language were introduced into the computational fluid dynamics (CFD) software to make the numerical results realistic. The thermal characteristics of the oscillatory thermal cycler chamber (OSTRYCH) under various operational conditions were numerically analyzed [23]. By introducing the thermal contact effect into a theoretical study and considering the thermal contact conductance coefficient, which is empirical by the experimental fitting, the experimental temperature profiles were compared well with the numerical simulations. Our group was the first group to introduce the thermal contact effect into a theoretical study that had been applied to the design of a PCR device. The PCR experiments presented that Hygromycin B DNA templates were amplified successfully [9]. In the present study, the RNA mixtures are pipetted in a fixed reaction chamber. The number of biological molecules adsorbed onto the channel or tube surface, and the PCR inhibition can be greatly reduced due to the fixed reaction chamber. Three aluminum heating blocks with individual thermal control modules are established along the pathway, and the DNA mixture inside the sample volume oscillates the pathway and is put in contact with three sequential isothermal zones. The CFPCR system holds the flexibility to change the reaction rate by changing the moving speed of the sample mixture. Two cartridge heaters are utilized within one aluminum block to improve the temperature uniformity. Low-cost heaters and a power supply system make our thermocycler easily portable. To reduce the thermal resistance effect between the heating block and the reaction chamber, thermal grease is applied to diminish the temperature difference between the thermal control sensor and the sample mixture. A more comprehensive investigation is conducted to speed up the reaction time by the arrangement of the heating blocks. An improved oscillating device is utilized to execute the extended function for RT-PCR. The micromilling chamber is oscillated by a servo motor and contacted with different isothermal heating blocks to successfully amplify the CDV templates. In the previous studies on miniaturized thermocycling devices, only a few researchers developed the oscillating thermocycler for RT-PCR. The present oscillating thermocycler combines the merits of the chamber type and the CF type systems. Miniaturized oscillating devices for RT-PCR with small sample adsorption and the compact thermal control module could be well-established in future markets.

## 2. Materials and Methods

**Figure 1:**
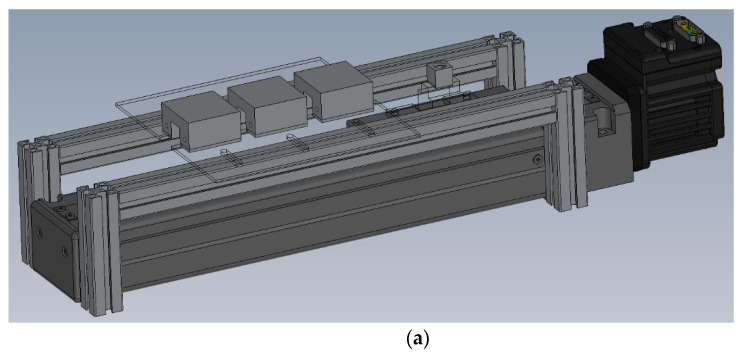

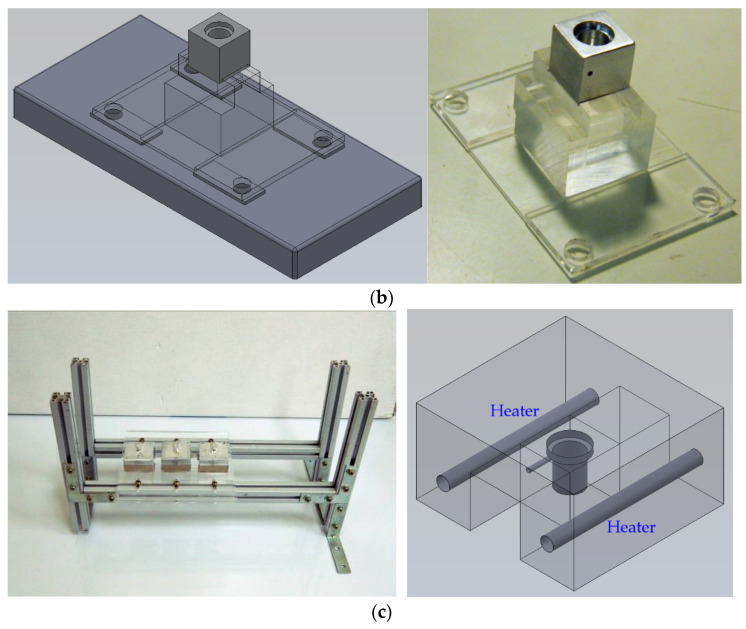
(**a**) The schematic diagram of the oscillating thermocycler. (**b**) The schematic diagram and the photograph of the aluminum chamber, PMMA block and sheet. (**c**) The photograph and schematic diagram of three heating blocks. Each consists of two heaters, one thermal sensor and an aluminum block.

The oscillating RT-PCR thermocycler is illustrated in Figure 1a. It consists of a rectangular reaction chamber with a cylindrical hole, three aluminum blocks with individual thermal control modules and a structural frame, some thermal sensors for sensing the temperatures, and a moving stage with a linear motor and a motion control system. The animation to illustrate the device working principle and handling is demonstrated in the Supplementary Materials, Video S1.

### 2.1. Design of the Reaction Chamber and Heating Blocks

The aluminum chamber, shown in Figure 1b, is fabricated utilizing a commercial computer numerical control (CNC) machine. The length, width, and height of the chamber are 12 mm, 12 mm, and 12 mm, respectively, and the geometric shape of the sample volume inside the reaction chamber has a diameter of 6 mm and a depth of 7 mm. A small hole with a diameter of 1 mm is drilled from the sidewall for the insertion of a thermocouple used for sensing the chamber temperature. The bottom of the chamber is glued onto the polymethylmethacrylate (PMMA) block, also shown in Figure 1b, which is immobilized onto the moving stage by a PMMA sheet. The PMMA block isolates the thermal energy from the reaction chamber and reduces the thermal damage of the moving stage. The RNA mixture is pipetted into the sample volume through the opening. Some mineral oil surrounds the reaction sample to prevent the sample from evaporation during RT-PCR.

Three aluminum blocks, expressed in Figure 1c, are of the same size. Two heaters are embedded in one heating block to ensure the thermal uniformity of the block. The length, width, and height of the block by its exterior are 38 mm, 36 mm, and 20 mm, respectively. Each block has a machined pathway with a cross-sectional area of 12 mm × 12 mm. The RNA mixture inside the sample volume oscillates the pathway and through three sequential isothermal zones, which correspond to the specific RT-PCR heating zones. The chamber is completely contained within a given isothermal zone. Before PCR, the reaction chamber moves to the Low Temperature (LT) zone to complete the RT stage, and then the LT zone is regulated to the annealing zone for the following PCR stage. The sequence of the isothermal zones can be changed for specific purposes. The three aluminum blocks are separated from each other by 12 mm air gaps which are long enough to avoid the thermal cross-talk between the adjacent heating blocks. These gaps are also served as the cooling zones. The total length of the moving displacement of the chamber is 98 mm. After a designated number of thermal cycles, such as 30~40 cycles, the sample mixture can be taken out for further gel electrophoresis.

### 2.2. Fabrication of the Thermal Control Modules

Some temperature difference exists between the sample mixture and the sensor for thermal control. With the purpose of achieving the required temperatures inside the sample volume during RT-PCR processes, the temperature difference has been measured to regulate the setting temperatures of the thermal control sensors before reactions.

Before the temperature regulations, the thermocouples are calibrated by measuring a series of fixed-point temperatures in a water bath (BH-130D, Yihder Co., Ltd., New Taipei, Taiwan). After reaching a steady-state temperature inside the bath, the temperatures of the thermocouples are recorded. The variations in the steady-state temperatures of the thermocouples are within the appropriate range of 0.3 K at three fixed temperatures of 55 °C (328 K), 75 °C (348 K), and 95 °C (368 K) in the water bath.

A thermocouple to measure the mixture temperature during the thermal cycling is inserted into the reaction volume through the opening of the cap, and the hole is sealed by acrylic adhesives. The measured temperatures are monitored using the thermocouples connected to a data acquisition system (Model NI 9211, National Instruments, Austin, TX, USA). The uncertainty of the temperature measured is ±1.17 K. The uncertainty of the temperature measured in our experiments ranges from 2.13% to 1.23% within the temperature range of 328 K to 368 K [24]. Then the temperature difference can be used for regulations.

**Figure 2:**
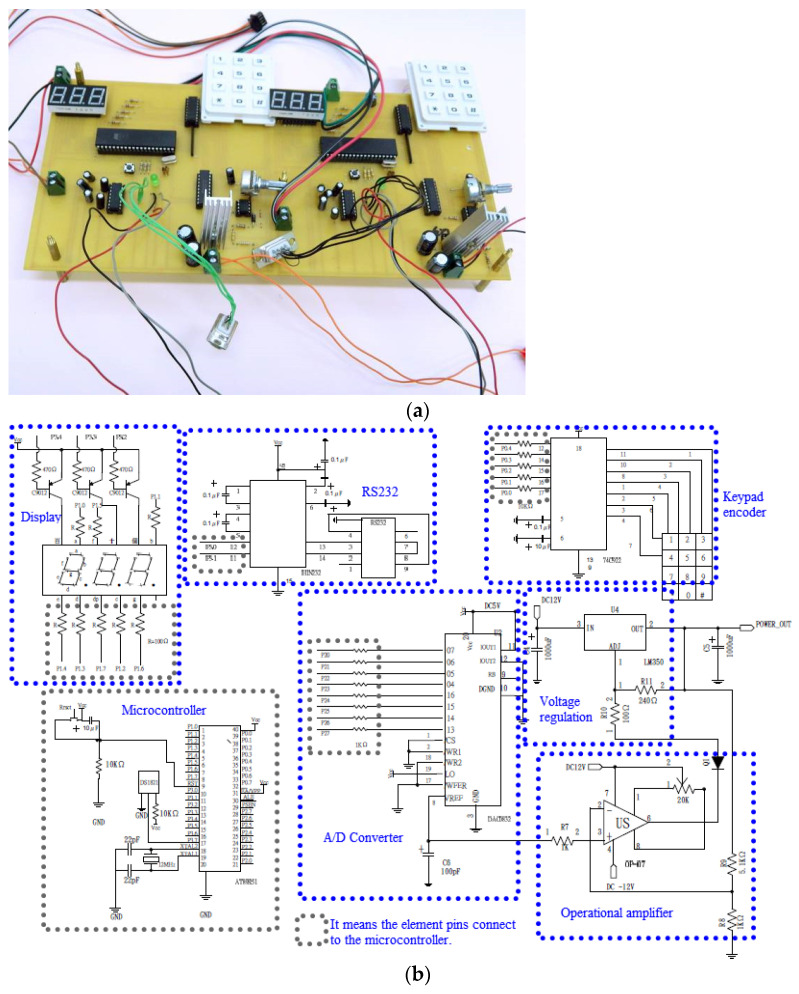

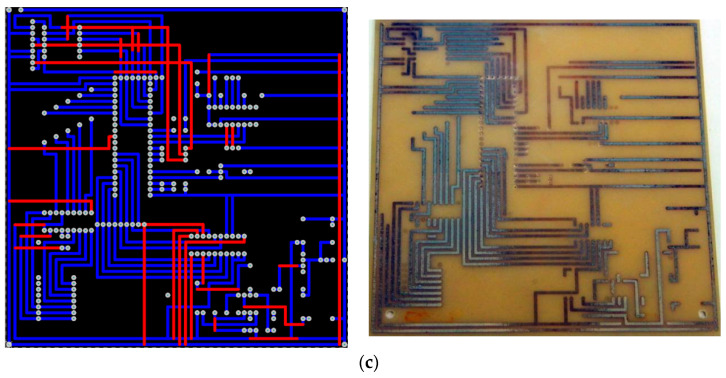
(**a**) The photograph of the homemade thermal control module. (**b**) The circuit diagram. (**c**) The schematic diagram and the photograph of the homemade PCB.

Thermal energy to RT-PCR processes is afforded with three heating blocks surrounding the chamber. The high thermal conductivity of aluminum confirms excellent temperature uniformity within each block. An individual block is equipped with two bores of 3.2 mm. Each bore houses one resistance cartridge heater (3.175 mm diameter, 38 mm length, 10 V, 14 W, C1J-9412, Watlow, St. Louis, MI, USA), and one K-type thermocouple (outer diameter of 0.254 mm, K30-2-506, Watlow, St. Louis, MI, USA). The thermocouple, which is attached to the surface of the cartridge heater, is connected to a homemade thermal control module, shown in Figure 2a. The control module receives the feedback signal from the thermocouple and determines the power input to the heater using a proportional/integral/derivative (PID) control scheme.

The circuits and the PCB layout are created using Design Explorer 99 SE software. The circuit diagram is illustrated in Figure 2b. The microcontroller in the PCB is an AT89S51 microcontroller (Microchip Technology Inc., Chandler, AZ, USA). To heat the aluminum reaction chamber, a voltage regulator IC (LM350) for direct current adjustable power supply is employed (Voltage regulation region shown in Figure 2b). Utilizing the operational amplifier (OP-07) and digital-to-analog (D/A) converter (DAC0832), the temperature value corresponding to the thermal sensor (DS18B21) is obtained (Operational amplifier and A/D converter regions shown in Figure 2b). A 3 × 4 keypad is decoded using a 74C922 keypad encoder IC and provides the user interface (Keypad encoder region shown in Figure 2b). The temperature of the aluminum block during the RT-PCR process is presented on a 7-segment light-emitting diode display (Display region shown in Figure 2b). The executable program is downloaded into the microcontroller through the serial port (RS232). In order to connect RS232 to the AT89S51 microcontroller, a converter is required. Here we make use of MAX232. This can convert the output of the microcontroller to the RS232 output level and vice versa.

Atmel Studio 7, an integrated development environment (IDE), is utilized for developing hardware offerings and applications. The ATmega328p-au microcontroller is programmed in the programming language, C++. The thermal sensor is connected to a homemade PCB, demonstrated in Figure 2c. The microcontroller receives the temperature signal and determines the power input to the heater using a PID algorithm. After the introduction of the sample, the heater is programmed to maintain a specific value (depending on the RNA mixtures).

A temperature drop is experienced at the interface between the heating block and the reaction chamber in contact [9]. The phenomenon resulting from the thermal contact resistance existing between the real contacting surfaces is diminished by applying some thermal grease. A small amount of thermal grease is applied between these contacting thermal elements to confirm the thermal contact. The temperatures of the DNA mixture with the setting points at 363 K, 322 K, and 335 K are 359.2 K, 326.82 K, and 337.7 K without thermal grease and 368.99 K, 331.3 K, and 346.58 K with thermal grease. To ensure the temperature uniformity of the aluminum blocks, the measured temperature difference of five different points on the surfaces of each block is within 2 K. The blocks are mounted on PMMA frames to thermally insulate each zone and certainly ensure the positional contact.

### 2.3. Programming of the Linear Motion System

The moving stage connected to the linear motor and microprocessor (SMART Motor SM2315D, Montrol Systems Co., Ltd., Taoyuan, Taiwan) drives the reaction chamber. The motor can be powered by a power supply ranging from 20 to 48 V. The bidirectional repeatability of the moving stage is less than 40 µm. The moving scenarios are programmable with the SmartMotor Interface software by setting the moving speeds, moving distances, moving times, and other related parameters. After the programming is finished and the code is transferred through an RS-232 port from the computer to the microprocessor, the motor can execute the code without the RS-232 connection.

### 2.4. Amplification of Canine Distemper Virus

Canine distemper virus (CDV) is a member of the genus *Morbillivirus* of the *Paramyxoviridae* family and causes an extremely contagious disease in carnivores and noncarnivores as well as marine mammals, but especially in dogs. On both the commercial PCR machine (MJ Mini™ 48-Well Personal Thermal Cycler, Bio-Rad, Hercules, CA, USA) and the oscillating thermocycler, a 385-bp segment of CDV RNA is amplified to evaluate the performance of the amplification. The sample mixture consists of 25 μL of 2× MyTaq One-Step mix (Bioline, Trento, Italy), 2 μL of both forward and reverse primer (10 μM), 0.5 μL of Reverse transcriptase, 1 μL of 10 U/µL RiboSafe RNase Inhibitor, and 5 μL of RNA template (35.4 ng/μL). DEPC-treated water is added up to the final volume of 50 μL.

The mineral oil is used to surround the DNA sample in the reaction volume, prevent the evaporation of the sample mixture, and reduce the wall adsorption of the reagent. The influence of various volumes of mineral oil on the sample temperature is investigated before RT-PCR. An aluminum block is heated to 357 K. The values of the ambient temperature, and relative humidity are about 299.5~300.2 K and 38~45%, respectively. The reaction chamber moves to the middle part of the heating block and stops for 90 s. The temperatures of 20 μL of DNA mixture rise from 300 K to 359 K, 358.3 K, 359.3 K, 359.4 K, and 358.1 K, with the mineral oil volume of 20 μL, 40 μL, 60 μL, 80 μL, and 100 μL. The temperature difference is within 1 K.

Before the sample injection, the bottom of the reaction chamber is covered with a 20-μL volume of mineral oil. The sample is then pipetted into the interior of the mineral oil. The DNA mixture is placed in a fixed chamber to reduce the problem of surface compatibility and to avoid inhibition of the PCR by interactions of biomolecules with the chamber walls. The peelable adhesive PCR film (Adhesive PCR Plate Seals, AB-0558, Thermo Fisher Scientific Inc., Waltham, MA, USA) is used for sealing the top of the chamber.

All three heaters are programmed to maintain the specific temperatures to perform RT-PCR. Subsequently, the annealing zone is maintained at 318 K (45 °C) for the reverse transcription. The whole system is insulated. After finishing the reverse transcription, the annealing zone is heated, and the PCR process begins. At the completion of 40 cycles, all the heaters are set to the extension temperature to facilitate the final step. Four thermocouples sensing the central temperature of the chamber and three heating blocks are connected to the NI 9211, which converts the analog signal to a digital one. A computer receives the temperature signals through the NI 9211 interface and records the real-time temperature profiles.

The thermal cycling program for the commercial PCR machine involves heating the mixture to 318 K for 20 min to execute the reverse transcription, 368 K for 1 min to activate the polymerase and denature the initial DNA, followed by thermal conditions consisting of denaturing at 368 K for 10 s, annealing at 332 K for 10 s, and extension at 345 K for 30 s. Upon completion of up to 40 thermal cycles, the chamber is kept at 345 K for 30 s for the final extension. The negative control experiment is conducted by replacing the template genomic DNA with nuclease-free water.

After the PCR process is finished, the products are collected from the chamber in a vial and mixed with 1× blue dye. The RT-PCR products are analyzed by agarose gel electrophoresis (Mini-Sub Cell GT System, Bio-Rad, Hercules, CA, USA). Ten microliters of each sample are loaded onto a 2% agarose gel (Certified Molecular Biology Agarose, Bio-Rad, Hercules, CA, USA) and electrophoresed in 10× Tris/Boric Acid/EDTA (TBE) buffer. The gel is run for about 40 min at 120 V. After electrophoresis, the gel is stained with 10 mg/mL ethidium bromide solution (Bio-Rad, Hercules, CA, USA) and imaged under UV illumination.

## 3. Results

An oscillating thermocycler is designed and fabricated for RT-PCR. To start with, various operational parameters on the temperature profiles of the designed device are demonstrated. Next, the accuracy, repeatability, and stability of the thermocycler on the thermal characteristics for PCR are expressed. Finally, the RT-PCR results of the canine distemper virus sample are shown in terms of gel electrophoresis.

### 3.1. Various Operational Parameters on the Temperature Profiles of the Reaction Chamber

In our oscillating thermocycler, the reactive sample is moved back and forth among three isothermal zones: Low Temperature (LT), Middle Temperature (MT), and High Temperature (HT) zones. The setting temperatures of LT, MT, and HT zones during the oscillatory motion are 322 K, 335 K, and 363 K, respectively. The moving speed is set to be 24 mm/s during the measurements. To simulate the DNA mixture during PCR, 20 μL of deionized (DI) water is placed into the chamber and surrounded by 20 μL of mineral oil for parametric tests. The above values of the parameters are used unless otherwise stated.

**Figure 3:**
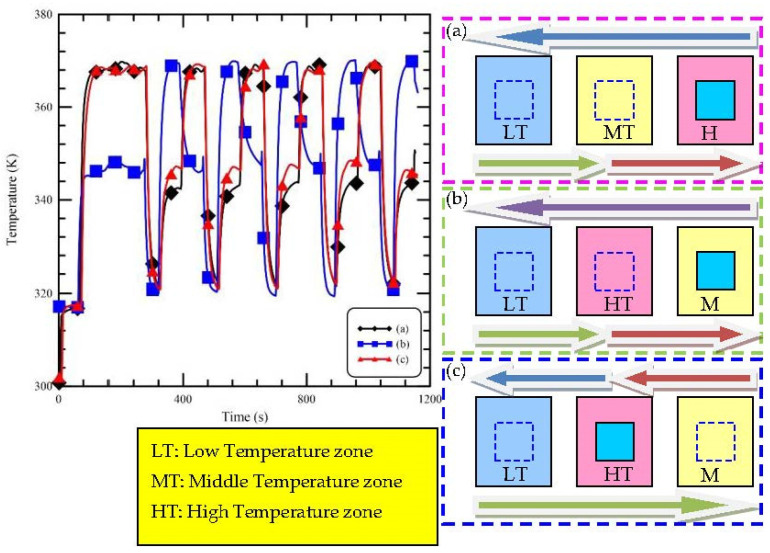
The influence of various arrangement of the heating blocks on the reaction chamber temperatures.

It depicts the schematic diagram of the oscillating thermocycler on the right side of Figure 3. The sample oscillates within a pathway integrated with a linear arrangement of isothermal zones. To investigate the heat transfer in the sample solution during the thermal cycles, the temperature profiles inside the reaction chamber influenced by the arrangement of the heating blocks are also demonstrated in Figure 3. The measured thermal sensor is inserted into the water inside the reaction chamber. During CFPCR, the temperature variation of the reaction mixture is dominated by the arrangement of heating blocks. The ramping rate is also enhanced by properly setting the arrangement. The heating blocks are assigned to be LT, MT, and HT zones from left to right, shown in Figure 3a. When the chamber moves near the HT zone, the temperature increases, and the chamber stays under the HT zone to reach a stable temperature for denaturation. Then the chamber continues on to the LT zone. The temperature drops to the annealing temperature. After that, the temperature increases gradually while the chamber moves back to the MT zone. As the temperature reaches the extension temperature for 30 s, the chamber is moved to the origin to start another thermal cycle. The chamber moves from the HT zone to the LT zone via the MT zone, as shown in Figure 3a. The energy transferred to the ambient from the reaction chamber occurs between the air gaps of two isothermal zones, but the chamber also receives the energy from the MT zone. There is not enough time for the chamber to be cooled down to the annealing temperature. After staying in the LT zone for a certain time, the chamber moves to the MT zone. It is found that the duration time for temperature rising is short, and the requested extension temperature is not reached. The arrangement of the heating zones is changed by switching the HT and MT zones in Figure 3b. The chamber starts from the MT zone, and the chamber temperature is maintained at a steady temperature for an extended time. Then the chamber moves to the LT zone via the HT zone. There exists a temperature peak as the chamber passes the HT zone and absorbs the energy from the HT zone. After that, the temperature is lowered to about 321 K for annealing. Next, the chamber changes location to the HT zone, and the temperature is leveled to about 368 K for denaturation. Though the chamber temperature reaches 368 K, 345 K, and 321 K, respectively, the temperature changes of the thermal cycles for a PCR process is not correct. In Figure 3c, the arrangement of the heating zones is the same as that in Figure 3b. The HT zone is the starting location for the chamber. Then the chamber moves to the LT zone and the MT zone in sequence. The chamber temperature changes from 368 K to 321 K and then 345 K. Then, it can be found as the chamber turns back from the LT zone to the MT zone via the HT zone. The temperature increases to a high value before entering the MT zone and the ramping rate is also increased due to the preheating effect. From Figure 3, it is found that after experiencing the HT zone for denaturing, the chamber moves to the LT zone for direct cooling, and a large ramping rate is acquired. Then, the chamber moves back to the MT zone via the HT zone, and the preheating effect increases the ramping rate. The arrangement of the heating blocks from left to right set to be the LT, HT, and MT zones, shown in Figure 3c, is suggested.

**Figure 4:**
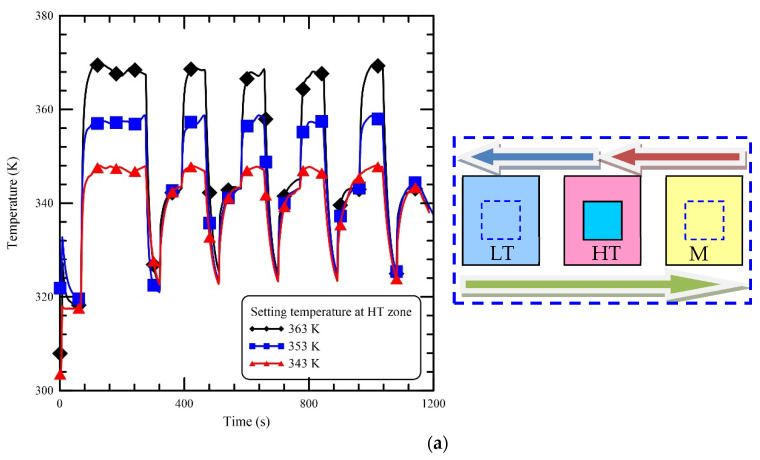

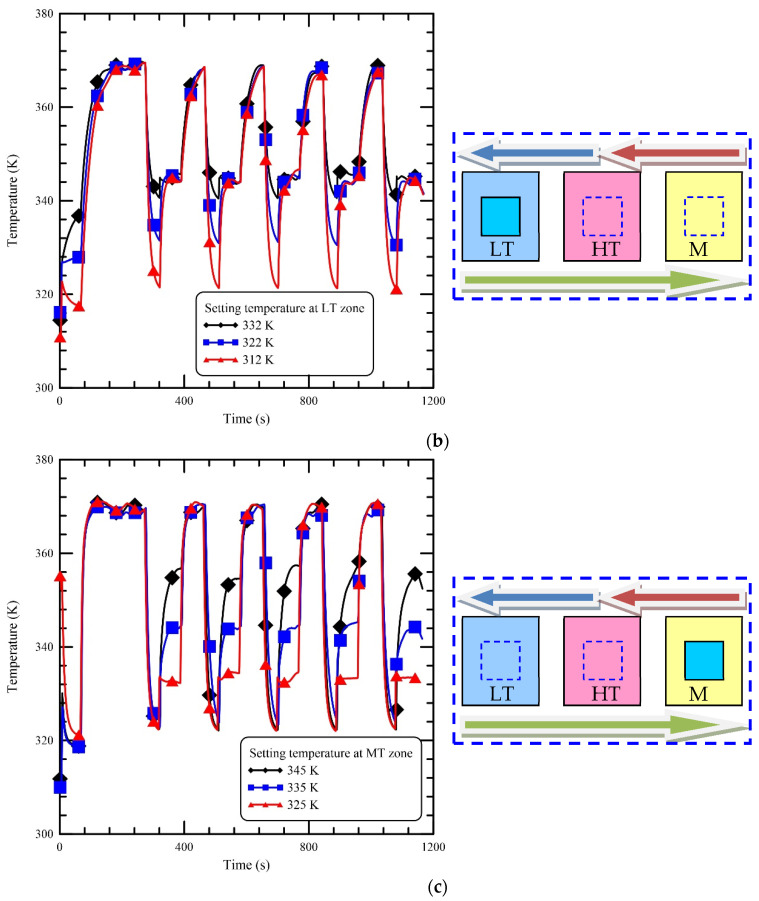
The influence of various heating temperatures of aluminum blocks on the reaction chamber temperatures. (**a**) The setting temperatures of the HT zone are 363 K, 353 K, and 343 K. (**b**) The setting temperatures of the LT zone are 332 K, 322 K, and 312 K. (**c**) The setting temperatures of the MT zone are 345 K, 335 K, and 325 K.

PCR cycling and operational parameters, such as the PCR temperatures and the duration times, have to be set up for effective amplification when appropriate amounts of DNA input and PCR components have been determined. The PCR temperatures at three stages for various DNA samples are not always the same. The setting PCR temperatures have to be suitable for specific DNA mixtures in order to be amplified efficiently. The influence of various heating temperatures of aluminum blocks on the mixture temperatures is presented in Figure 4. The setting temperatures of the HT zone are 363 K, 353 K, and 343 K, respectively, in Figure 4a. It is found that the time required to reach the setting temperatures in the HT zone is almost the same. After passing the HT zone, the reaction chamber moves to the LT, and the time to achieve the annealing temperature increases as the temperature of the HT zone is high. Then the chamber moves to the MT zone via the HT zone, and the rising time decreases due to the larger preheat effect of the HT zone at 363 K than the others. The temperatures of the DNA mixture at the LT and MT zones are not affected by the setting temperatures of the HT zone. Figure 4b shows the effect of various setting temperatures at the LT zone on the temperature profiles of the reaction chamber. The setting temperatures of the LT zone are 332 K, 322 K, and 312 K, respectively. The temperatures of the reaction chamber at the HT zone are definitely unaffected by the setting temperatures at the LT zone. The steady temperatures of the chamber at the MT zone show some difference among these cases. The chamber temperature reaches the annealing temperature at the LT zone, and it moves to the MT zone after passing the HT zone as well as absorbing the high thermal energy. For the case of 332 K, the chamber temperature increases to a higher value than the setting temperature at the MT zone and then lowers to the setting temperature at the MT zone. However, the difference among these cases in Figure 4b is not obvious. The block temperature at the MT zone is set at 345 K, 335 K, and 325 K, respectively, as shown in Figure 4c. The rising time to reach the setting temperature is longer as the temperature of the MT zone is higher. For the CFPCR, the reaction mixture moves through the individual isothermal zones to finish the reaction stages. It is necessary for the heating blocks to maintain a constant temperatures during the reaction. It is no doubt that there is no significant influence on the chamber temperatures as any setting temperature changes in our oscillatory thermocycler. Therefore, heat management without thermal cross-talk is easy with our device.

**Figure 5:**
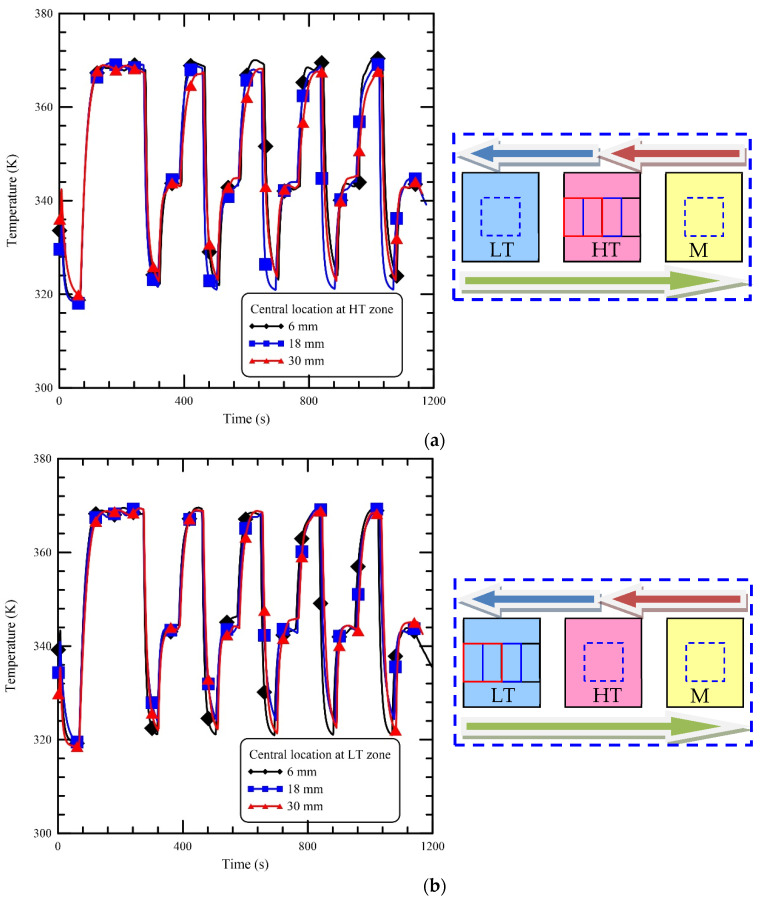

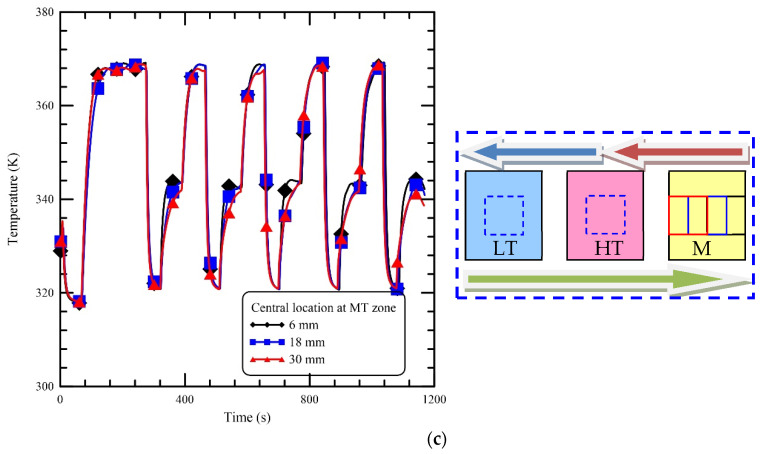
The influence of various chamber locations on the reaction chamber temperatures. (**a**) The temperature profiles of the chamber staying at the locations of the HT zone of 6 mm, 18 mm, and 30 mm. (**b**) The temperature profiles of the chamber staying at the locations of the LT zone of 6 mm, 18 mm, and 30 mm. (**c**) The temperature profiles of the chamber staying at the locations of the MT zone of 6 mm, 18 mm, and 30 mm.

The high thermal conductivity of aluminum blocks ensures the temperature uniformity of the heating blocks. The energy conducted from the heating block to the reaction chamber is similar when the volume of the chamber is located within the heating zone. However, the heat transfer at the front and back surfaces of the reaction chamber that are exposed to the ambient is dominated by natural convection. The boundary conditions for the chamber at various locations are different. The influence on the mixture temperature at various chamber locations is demonstrated in Figure 5. The highest temperatures of the chamber staying at the locations of the HT zone of 6 mm, 18 mm, and 30 mm, respectively, are 369.6 K, 368.7 K, and 367.1 K in Figure 5a. The temperature difference is within 2.5 K. From Figure 5b, it is found that the lowest temperatures are 321.2 K, 324.1 K, and 322.2 K at the locations of the LT zone of 6 mm, 18 mm, and 30 mm, respectively. The temperature difference is within 2.9 K. The maximum temperatures of the chamber at the MT zone are 343.6 K, 343.3 K, and 342.9 K at the locations of 6 mm, 18 mm, and 30 mm, respectively. The temperature difference is within 0.7 K, shown in Figure 5c. In our oscillatory thermocycler, the temperature differences at HT, LT, and MT zones are within 2.5 K, 2.9 K, and 0.7 K, respectively, at different locations. The temperature variations of the chamber at various locations under the specific heating zones can be negligible. To reduce the temperature variations due to the unexpected change in the ambient thermal conditions, the chamber always moves to the central location (18 mm) under the individual heating zone.

**Figure 6:**
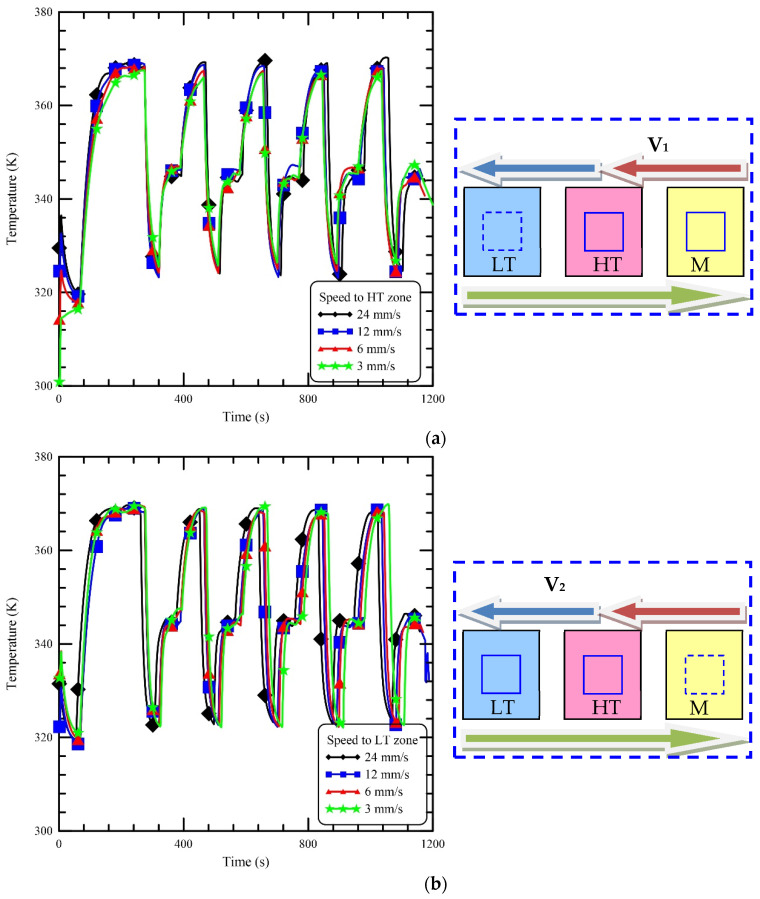

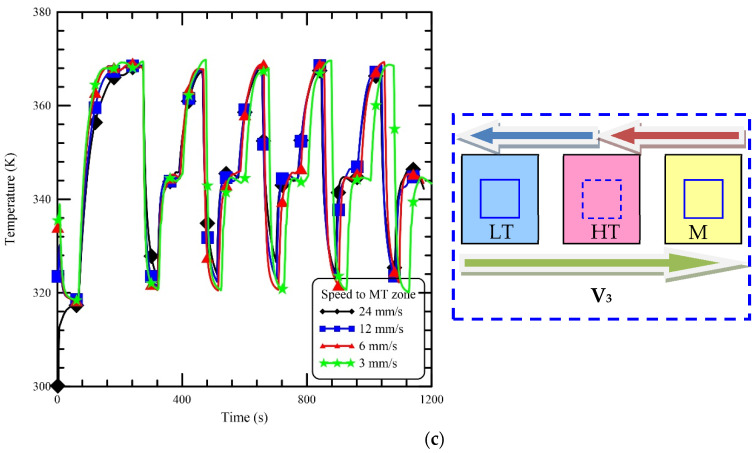
The influence of various travelling speeds on the reaction chamber temperatures. (**a**) The temperature profiles of the chamber travelling from the MT zone to the HT zone at the speeds of 24 mm/s, 12 mm/s, 6 mm/s, and 3 mm/s. (**b**) The temperature profiles of the chamber travelling from the HT zone to the LT zone at the speeds of 24 mm/s, 12 mm/s, 6 mm/s, and 3 mm/s. (**c**) The temperature profiles of the chamber travelling from the LT zone to the MT zone at the speeds of 24 mm/s, 12 mm/s, 6 mm/s, and 3 mm/s.

The ramping rate of the thermocycling process is one of the most important factors in the shortening of the reaction time. During the CFPCR process, the reaction chamber moves to the specific reaction zones to complete PCR. The ramping rate is influenced by the travelling speed. The effect of various travelling speeds on the reaction chamber temperatures is demonstrated in Figure 6. The moving speed of the reaction chamber changes from 24 mm/s, 12 mm/s, 6 mm/s to 3 mm/s, and the travelling time from the MT zone to the HT zone are 2 s, 4 s, 8 s, and 16 s, respectively, in Figure 6a. The effect of various moving speeds from the MT zone to the HT zone on the chamber temperature is presented. Short travelling time means long heating or cooling time for the reaction chamber. The maximum chamber temperatures for different speeds are 369.3 K, 368.7 K, 367.4 K, and 366.1 K, respectively, shown in Figure 6a. The temperature difference is within 3.2 K. The maximum difference in the duration time among these cases at every cycle is 14 s. The moving speed from the HT zone to the LT zone is from 24 mm/s to 3 mm/s shown in Figure 6b. The lowest temperatures for four cases range from 322.2 K to 322.8 K, and within 0.6 K. The travelling distance from the LT zone to the MT zone is two times larger than the cases presented in Figure 6a,b. Since the chamber moves from the LT zone to the MT zone via the HT zone, the preheating during the travelling via the HT zone makes the chamber temperature reach the required extension temperature after entering the MT zone quickly. We can find that the temperature difference among these cases is less than 1.5 K. As the chamber speed is decreased from the MT zone to the HT zone, the reaction time delay is obvious. So, the moving speed at 24 mm/s is used as our operational parameter.

### 3.2. Thermal Characteristics of the Reaction Chamber during PCR

During the PCR process, the accuracy of the reaction temperature, stability, and repeatability of the thermal characteristics during PCR are essential for finishing an effective amplification process. A standard deviation of several consecutive temperature measurements is used to present the thermal characteristics of our oscillating thermocycler. The operational parameters are the same as those in the previous cases.

**Figure 7:**
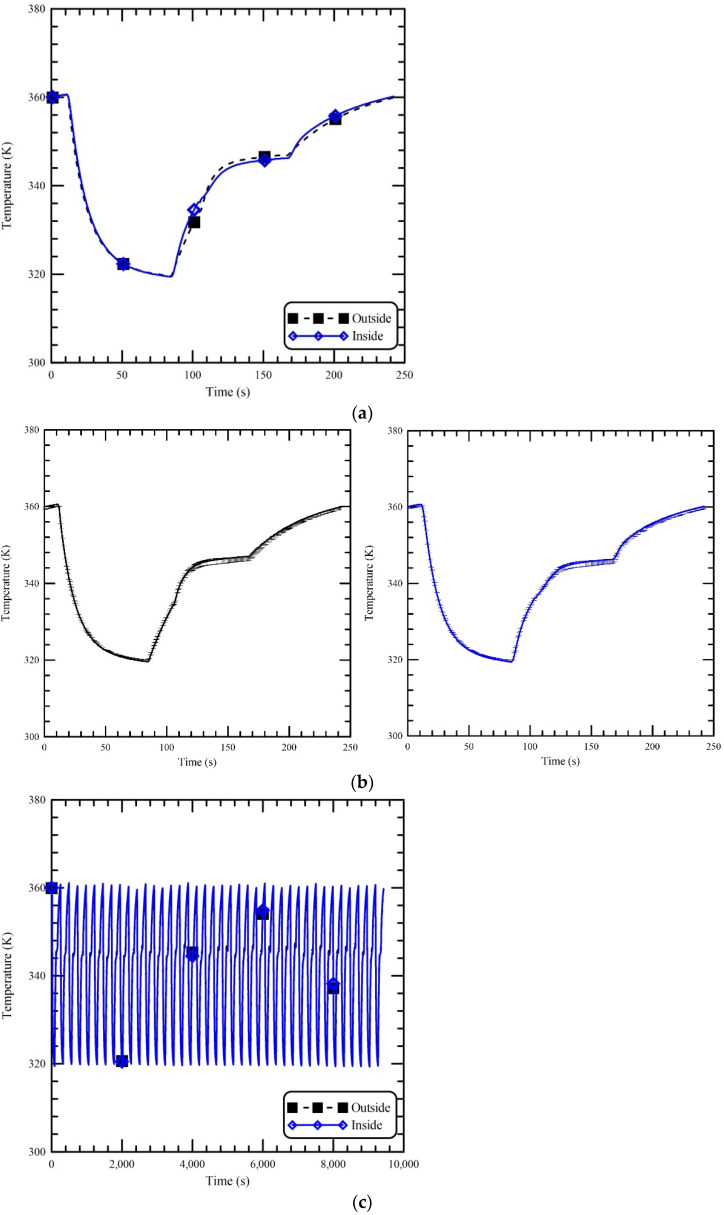
(**a**) The accuracy, (**b**) the repeatability and (**c**) the stability of the thermal characteristics are examined in our oscillating thermocycler.

The accuracy, repeatability, and stability of the thermal characteristics are examined in our oscillating thermocycler and demonstrated in Figure 7. The thermal cycling profiles detected by the thermocouple outside and inside the chamber on different days are investigated, and one cycle is shown in Figure 7a. The average temperatures at four sets of measurements are demonstrated. Results show that the temperature curve inside the chamber is similar to that outside the chamber. The temperature profile is accurately suitable for the PCR process. The repeatability of the oscillating thermocycler is established in Figure 7b. The thermal cycling profiles detected by the thermocouple outside (left) and inside (right) the chamber for 40 cycles are also shown. It demonstrates that the temperature curves of the chamber for 40 times are similar; the standard deviations of the temperatures at 40 sets of measurements are within 1.339 K. Then, the repeatability of this oscillatory thermal cycler chamber is then confirmed. The stability of the temperature cycles is also expressed in Figure 7c. The measured temperature profiles outside and inside the chamber of a forty-cycle reaction are investigated. Very little difference between the temperature profiles of each consecutive cycle is exhibited. It verifies the stability of the oscillatory thermocycler chamber during PCR.

### 3.3. Gel Electrophoresis Results for CDV Samples

**Figure 8:**
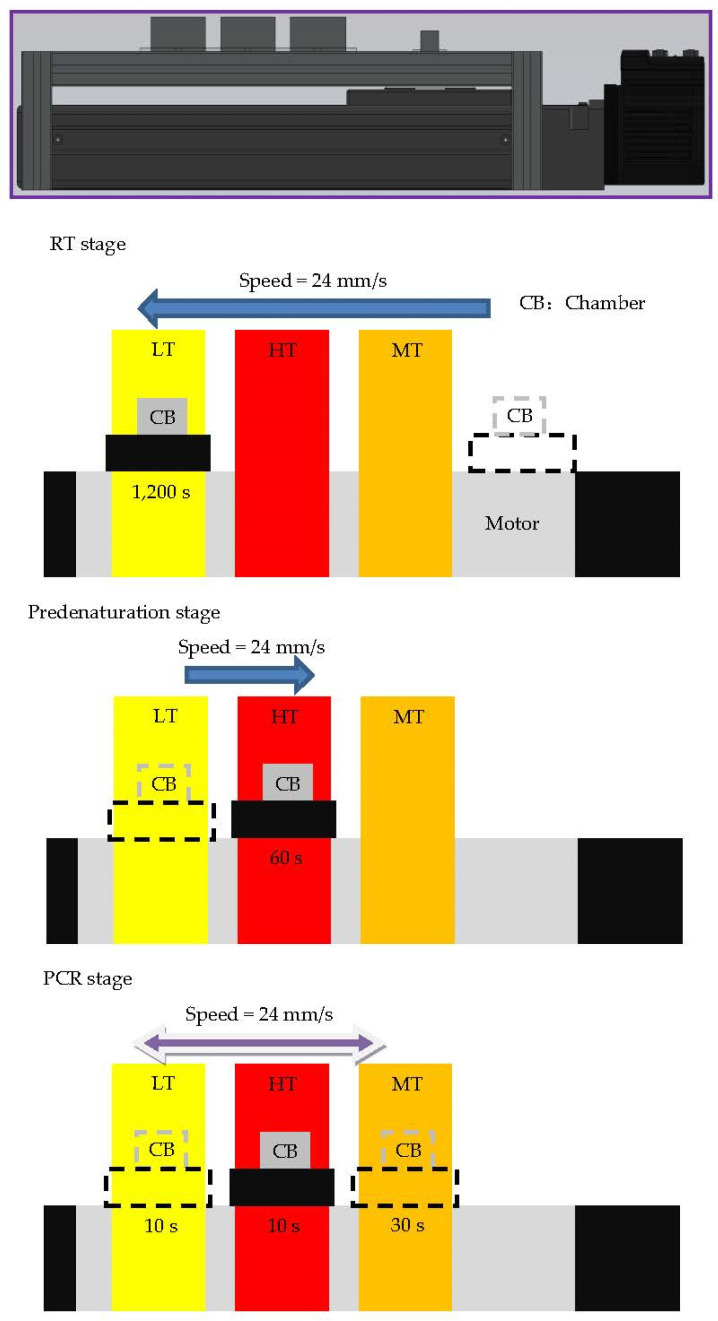
The physical configuration of the oscillating thermocycler. Three stages including RT, predenaturation, and PCR stages, are illustrated.

The integration of RT and PCR is performed on the oscillating thermocycler. The arrangement of the heating blocks from the left to right set to be the LT, HT, and MT zones is utilized and depicted in Figure 8. The moving speed of the reaction chamber is 24 mm/s during RT-PCR. The RT-PCR with RNAs from CDV strains results in amplicons of length 385 bp. Before PCR, the reaction chamber stays at the LT zone to complete the RT stage, and then the LT zone is regulated to the annealing zone to continue the PCR stage.

**Figure 9:**
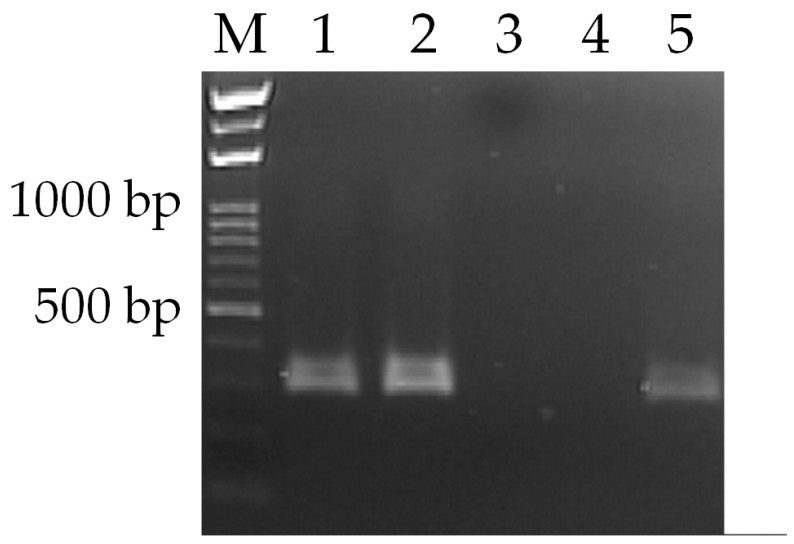
Agarose gel electrophoresis of the RT-PCR yield. Lane (M): DNA ladder; lanes (1 and 2): positive control from the conventional PCR machine; lane (3): negative control from the conventional PCR machine; lane (4): negative control from the oscillating thermocycler; lane (5): positive control from the oscillating thermocycler.

Two protocols are used for performing RT-PCR on the oscillating thermocycler in the following. In protocol A, the RT reaction mixture is moved into the LT zone. At this location, the RT is allowed to incubate for 20 min at 318 K. Following RT, the sample is manually transferred to the commercial PCR machine for a PCR process. All products were collected at 40 cycles. In protocol B, the reaction chamber stays at our thermocycler to complete the RT stage. Then the PCR stage is subsequently performed in our oscillating thermocycler. Figure 9 shows the sample mixture of the RT-PCR process on the gel electrophoresis results of the amplification product. M defines DNA markers and indicates the DNA ladders. Results clearly present the 385-bp sample product in the commercial PCR machine (Lanes 1 and 2). The PCR stage is performed in our oscillating thermocycler, and the result also presents the 385-bp product (Lane 5). Negative controls show no DNA templates in the commercial PCR machine and our thermocycler at Lanes 3 and 4, respectively. The results from the oscillating thermocycler are comparable to those obtained by the commercial PCR machine. In our oscillating thermocycler, the RT and RT-PCR processes are confirmed in Figure 9.

**Figure 10:**
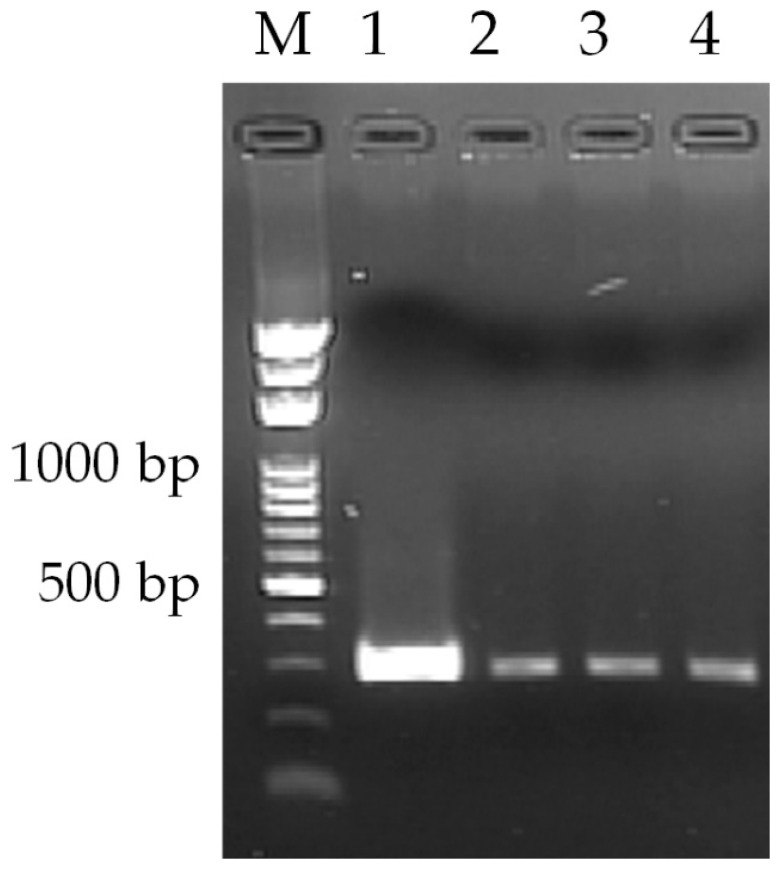
Agarose gel electrophoresis of the RT-PCR yield. Lane (M): DNA ladder; lane (1): positive control from the conventional PCR machine; lanes (2~4): RT-PCR products from the oscillating thermocycler using various volumes of mineral oil for 30 μL, 20 μL, and 10 μL, respectively.

The amount of mineral oil used in the RNA mixture can ensure that the RT-PCR amplification is successfully carried out without the sample evaporation. The continuous-flow amplification device is performed using various volumes of mineral oil for 30 μL, 20 μL, and 10 μL, respectively. Figure 10 displays the effects of various mineral oil volumes on RT-PCR amplification when the sample mixture is driven to move back and forth through the pathway. In the conventional PCR machine, the reactions are performed effectively with 10-μL of mineral oil (Lane 1). In the oscillating thermocycler, the sample mixtures do produce the anticipated number of products (30, 20, and 10-μL of mineral oil at Lanes 2, 3, and 4). The measured sample temperature difference is within 1 K with the mineral oil volume of 20~100 μL. There is little discrimination among the results of Lanes 2~4.

## 4. Conclusions

Since PCR was invented, it has emerged as a powerful tool in genetic analyses. The PCR products are closely linked with temperature cycling. In order to reduce the reaction time and make the temperature distribution uniform in the reaction chamber, we design an oscillating thermocycler for RT-PCR. In this paper, we suggest the denaturation zone located between the annealing and extension zones as the proper arrangement for the heating blocks on the thermocycler. The chamber moving speed of 24 mm/s is utilized to reduce the reaction time. The experimental result indicates that the effects of various chamber locations under the heating block on temperature uniformity are negligible. To verify the temperature repeatability, the standard deviations of the temperatures at 40 sets of measurements are within 1.339 K. Finally, an improved oscillating device is demonstrated to amplify the canine distemper virus templates. The total length of the moving displacement of the chamber is 98 mm. By properly selecting a linear moving stage, the reported platform will be miniaturized, and the length, width, and height of the device in appearance are within 200 mm, 200 mm, and 200 mm, respectively. There is enough space between the MT and HT zones for setting up an optical detection module, e.g., a Raspberry Pi camera module. In our work, the developed real-time detection device will cost less than $1000 USD. The unique architecture utilized in this device is well applied to a low-cost DNA analysis system. The medical resources are not enough in remote areas. The laboratory work cannot be supported immediately, and then the low-cost detection system is the appropriate way to solve the difficult situations. Point-of-Care (PoC) diagnostics are commonly based on portable, inexpensive, and user-friendly sensor platforms and allow sensitive, robust, and real-time detection of biotargets. The major goals of this paper are to investigate the physical insights of the thermal characteristics and the amplification performances in the present oscillating thermocycler. Our future work will be focused on a miniature light-emitting diode (LED)-induced fluorescence detection system with our oscillating thermocycling system. Then the amount of amplification at specific cycle numbers can be quantified. The sensitivity and the specificity of the fabricated system will be confirmed.

## Supplementary Materials

The following supporting information can be downloaded at: https://www.mdpi.com/article/10.3390/mi13040600/s1, Table S1: The comparison of important technical aspects with previous reports; Video S1: The animation to illustrate the device working principle and handling.
