# Supplementary material for: Fabrication of an Oscillating Thermocycler to Analyze the Canine Distemper Virus by Utilizing Reverse Transcription Polymerase Chain Reaction
Source: Micromachines (Basel). 2022 Apr 12;13(4):600. doi: 10.3390/mi13040600 (PMC9026093; doi:10.3390/mi13040600)
Supplement: Supplementary file 1 [file micromachines-13-00600-s001.zip › Supporting Information Table S1.pdf]

Fabrication of an oscillating thermocycler to analyze the canine distemper virus by utilizing reverse transcription polymerase chain reaction

Jyh Jian Chen [1],* and Zong Hong Lin [2]

1 Department of Biomechatronics Engineering, National Pingtung University of Science and Technology 1; chaucer@mail.npust.edu.tw

2 Department of Biomechatronics Engineering, National Pingtung University of Science and Technology 2; o7392618@yahoo.com.tw

* Correspondence: chaucer@mail.npust.edu.tw; Tel.: +886-8-770-3202 (ext. 7029)

Table S1. The comparison of important technical aspects with previous reports.

Heater type	Thermocycling type	Continuous flow type	Cited references
commercial machine	stationary chamber		1~6, 16~17
infrared laser	stationary chamber		14
infrared heater	stationary chamber		15
thin film heater	continuous flow	Unidirectional (syringe pump)	8
cartridge heater	continuous flow	Unidirectional (syringe pump)	9
hot plate	continuous flow	Unidirectional (syringe pump)	10
PCB heater	continuous flow	Closed (stepper motor)	11
thin film heater	continuous flow	Closed (buoyancy)	12
cartridge heater	continuous flow	Oscillatory (servo motor)	7, 21
thin film heater	continuous flow	Oscillatory (dielectrophoresis)	13
infrared heater	continuous flow	Oscillatory (servo motor)	22
cartridge heater	continuous flow	Oscillatory (servo motor)	current work
